# Dynamic Compressibility of Poly (Vinyl Acetate) and Its Relation to Free Volume

**DOI:** 10.6028/jres.067A.007

**Published:** 1963-02-01

**Authors:** John E. McKinney, Harriet Vera Belcher

## Abstract

The complex bulk compliance (dynamic compressibility) of a commercial sample of poly (vinyl acetate), AYAT, was measured at frequencies from 50 to 1,000 cycles per second, temperatures from 0 to 100 °C, and static hydrostatic stresses from 0 to 981 bars (gage pressure) using an alternating hydrostatic stress generated and detected by piezoelectric transducers mounted in an essentially noncompliant cavity with dimensions small in comparison to a wavelength. The above temperature range was more than sufficient to cover the dispersion region in which an inflection in the storage compliance and a maximum in the loss compliance were observed. The data were reduced to functions of reduced variables using the WLF Equations extended to include static pressure with the “universal” WLF Constants and *dT/dP* = 0.020 °C/bar. The difference in limiting compliances at zero and infinite frequencies was larger than that predicted from the *dT/dP* shift using the free volume concept. A discussion is presented on possible processes that might contribute to an excessive value between limiting compliances.

## 1. Introduction

The free volume concept [1] 1 has been used frequently to explain the glass transition of polymers and glass forming liquids. To apply this concept it is postulated that the necessary and sufficient condition for a glass transition to occur in a polymer is that the free volume fraction reach the critical value necessary for continuously changing molecular arrangements, which account for the physical behavior typical of the liquid or rubbery state. This critical value of free volume fraction has been assumed to be independent of temperature and static pressure for a given material and nearly independent of the material (as long as it will undergo a glass transition under some conditions of state).

Measurements of the dynamic compressibility of vulcanized natural rubber-12 percent sulfur [2] were interpreted in terms of this concept, assuming that the compressibility observed at frequencies above (or temperatures below) the frequency- dispersion region corresponded to the compressibility of the occupied volume alone, and that the compressibility at frequencies below (or temperatures above) the dispersion region corresponded to the compressibility of the occupied plus the free volume. An extended WLF [1] equation including a linear dependence of free volume fraction on static pressure was used to reduce these observations to functions of a single variable. The “universal” WLF constants were found adequate to reduce the results at one atmosphere in terms of a single variable including frequency and temperature. The coefficient of pressure in the free volume fraction expression, evaluated empirically from the shifting of the dispersion region due to pressure, agreed with the isothermal compressibility difference calculated from the adiabatic difference in compressibility above and below the dispersion region.

For rubber-sulfur the shift in temperature with static pressure at constant free volume, 0.024 °C/bar, was nearly the same as that found for several other polymers investigated by different techniques: 0.025 for polyisobutylene [3], 0.027 for polyethylene [4], and 0.031 for polystyrene [5] hinting at the possibility that these numbers might be approximated by a “universal” constant as in the case of the WLF Universal Constants.

The purpose of this work was to investigate the effect of static pressure, temperature, and frequency on the dynamic compressibility of poly (vinyl acetate) in the vicinity of the glass transition in order to check on a different polymeric system the applicability of this free volume concept to explain the effects of pressure on mechanical properties and to evaluate the applicable constants.

## 2. Experimental Method

The dynamic bulk compliance^2^ measurements were made with an apparatus described in previous publications [6, 7]. In this method the sample is contained, along with two PZT 3 ceramic volume expander transducers mounted on compliant copper springs, within a cylindrical cavity sufficiently small to make the acoustic pressure hydrostatic. The remaining air is displaced with a pressure transmitting fluid [*di*(2 ethyl hexyl) sebacate] whose adiabatic compressibility is known from previous PVT and heat capacity measurements. An a-c voltage is applied to one transducer and a corresponding signal is observed on the other. From the complex voltage ratio the complex bulk compliances may be calculated. The temperature is controlled by immersing the entire assembly in a thermostated bath and any desired static pressure in the range of 0 to 1,000 bars (gage pressure) may be superimposed upon the dynamic pressure using a hand operated pump.

The calibration procedure and the dynamic bulk compliance determinations on poly (vinyl acetate) were similar to those used on rubber sulfur [2]. All runs were made along an isobar with increasing temperature and the system was permitted to reach thermal equilibrium before each reading was recorded.

It is required that the specific volume of the sample be known as a function of temperature and pressure in order to calculate its dynamic bulk compliance from the transducer equations. Also such data yield useful information including the thermal expansivity, the isothermal compressibility, the glass transition temperature and its shift with pressure, all of which are useful in evaluating the parameters for a reduced variable formulation.

The specific volume of this sample of poly(vinyl acetate) was determined by using a glass dilatometer with mercury as a confining liquid. This was placed in a pressure cell with glass windows to view the height of the mercury column. The cell in turn was placed in a liquid thermostat. The pressure was limited to 500 bars to prevent collapse of the glass windows.

## 3. Samples

The poly (vinyl acetate) supplied through the courtesy of G. M. Powell of the Bakelite Company was a commercial sample designated AYAT for which the intrinsic viscosity in cyclohexanone at 20 °C was given as 0.69 dl/g. The polymer was received in the form of small pellets averaging about 0.6 cm in diameter. These were molded at about 100 °C into disks 1.5 cm in diameter and 3 mm thick. In order to determine if there were any significant swelling in the pressure transmitting oil [*di*(2 ethyl hexyl) sebacate], one of the disks was placed in the oil and weighed at approximately weekly intervals for six weeks. All of the weighings agreed within 2 parts in 10^4^ indicating no significant swelling which could influence the bulk compliance determinations. In addition, the disk used in the bulk compliance determinations was weighed at frequent intervals to check for any effect of pressure on the swelling rate. Again the weighings agreed to within 2 parts in 10^4^.

## 4. Results

### 4.1. Specific Volume Measurements

**Figure 1:**
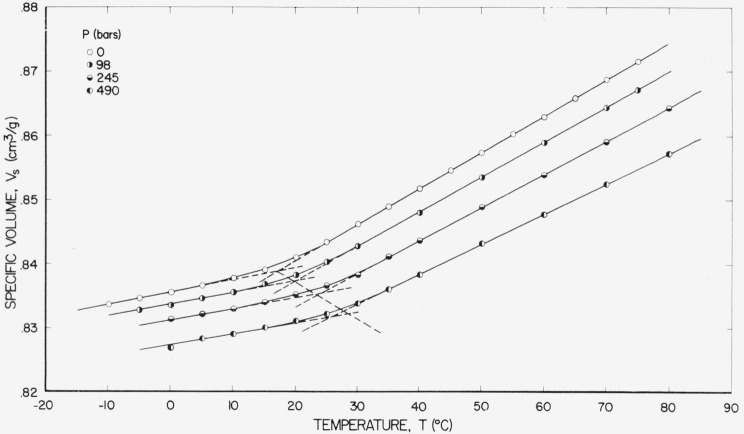
Specific volume of PVAc plotted against temperature at several pressures.

The specific volume, *V*_s_, of PVAc as a function of temperature at several static pressures is shown on figure 1. All of the observations were taken along an isobar for each of the four runs. Measurements in the liquid region were independent of the direction of temperature change and reached equilibrium in about 15 min, which is about the time for the apparatus to reach equilibrium. In the glassy region about 1 hr was required to obtain apparent equilibrium^4^ for each observation at constant pressure. It was found that the most reproducible measurements could be taken by commencing at some temperature above the transition region and then decreasing the temperature at constant pressure, allowing sufficient time to reach apparent equilibrium for each observation.

The dashed lines with positive slopes are extrapolated values of *V_l_* the specific volume established from volume-temperature relations in the liquid state, and *V_g_*, established from volume-temperature relations in the glassy state. *V_l_* and *V_g_* were obtained by a least squares fit on the data, linear in *T* and quadratic in *P*, treating the liquid and glassy regions separately and ignoring the data in the transition region. The glass transition temperature, *T_g_*, was obtained from the intersections of the extrapolations of *V_l_* and *V_g_* and was found to be 17 °C at 1 atm (*P*=0) and vary with pressure at a rate of about 0.020 °C/bar, as indicated by the dashed line with negative slope. As seems characteristic of poly (vinyl acetate), the glass transition temperature is poorly defined, since the thermal expansivity changes markedly over a temperature range of about 15°.

We had originally thought that our sample was a pure polymer, free of contaminant or very low molecular weight components; however, the value of *T_g_* determined from this experiment is low in comparison with the generally accepted values of 25 to 32 °C. The low value obtained here may be partly attributed to the longer than usual times permitted for the specific volume observations to approach apparent equilibrium. On the other hand heating the sample in a vacuum at 70 °C for one week resulted in a loss of weight amounting to almost 2 percent. The presence of contaminant could lower the value of *T_g_* and therefore contribute to the lower than usual value observed; thus, the data from our sample do not apply to pure PVAc, but to a sample with a small amount of contaminant. This uncertainty does not, of course, affect the validity of our results so far as our primary purpose is concerned.

Two additional thermodynamic quantities that are sometimes useful in evaluating some of the reduced variable parameters are the isobaric thermal expansivity,

$$\alpha =\frac{1}{V}{\left(\frac{\partial V}{\partial T}\right)}_{P},$$

and the isothermal compressibility,

$$\beta =-\frac{1}{V}{\left(\frac{\partial V}{\partial P}\right)}_{T}.$$

The related reduced variable parameters are supposed to be the differences in the values of *α* and the values of *β* between the liquid and glassy states. Table 1 gives the thermal expansivities for the liquid state,

$$\alpha_{l}=\frac{1}{V_{l}}{\left(\frac{\partial V_{l}}{\partial T}\right)}_{P},$$

for the glassy state,

$$\alpha_{g}=\frac{1}{V_{g}}{\left(\frac{\partial V_{g}}{\partial T}\right)}_{P},$$

and the difference, Δ*α*, evaluated at *T=T_g_* for each observed pressure.

The compressibility, *β*, was difficult to determine due to the lack of data at different pressures, in particular those above 500 bars; hence, any attempted evaluation to obtain the desired difference, Δ*β*, will be too uncertain to have any merit.

Values of *V_s_* at pressures higher than 490 bars required for dynamic compressibility determinations (735 and 981 bars) were evaluated by linear extrapolation using slopes determined from values at 245 and 490 bars. Although the dependence of *V*_s_ on *P* is not exactly known at the higher pressures, this procedure is legitimate because of the small change in *V_s_* with *P.* For example, an error in the slope of 10 percent will only produce a 0.2 percent error in *V_s_*, which is smaller than other uncertainties involved.

### 4.2. Dynamic Bulk Compliance Measurements

**Figure 2:**
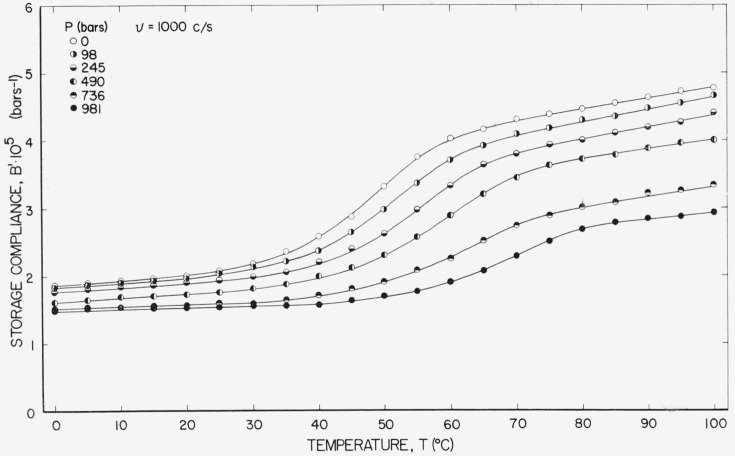
Storage compliance at 1,000 c/s plotted against temperature at several pressures.

Figure 2 shows the storage compliance, *B*′, at 1,000 c/s as a function of temperature at different static pressures. At the higher pressures the data for some of the runs appear to be biased. That is, all of the values at one pressure appear to be in error by nearly a constant amount over a large temperature range. For example, the lower four curves were taken at equal pressure intervals; however, the compliance values, especially at high temperatures, do not decrease in a smooth fashion with pressure as would be expected. In particular the gap between the 490 and 736 bar curves appears to be excessive. This effect is attributed to variations in transducer response between the sample and calibration runs. The larger the temperature and pressure ranges that are covered, the less reproducible the measurements become, presumably because of irreversible changes in domain configuration within the transducers. For an isothermal run at 90 °C the response of *B'* with pressure was much smoother and, in particular, the excessive gap between the values of *B'* at 490 and 736 bars shown in figure 2 was not apparent.

**Figure 3:**
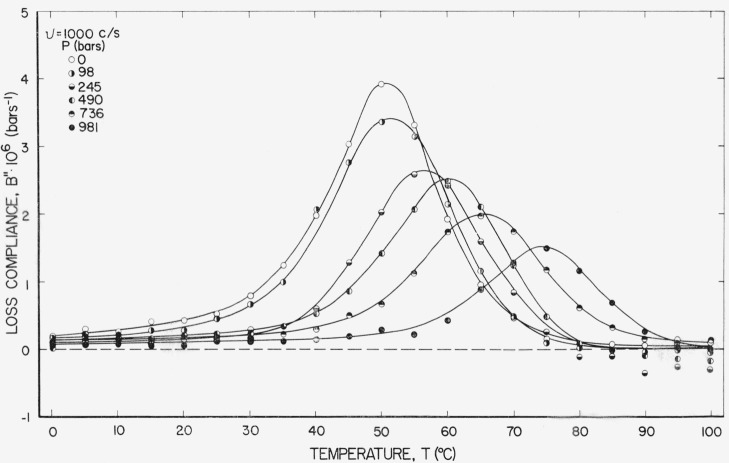
Loss compliance at 1,000 c/s plotted against temperature at several pressures.

Figure 3 shows the corresponding loss compliance. The temperatures for maximum *B*″ appear nearly the same as those for the inflections in *B*′ in figure 2. For a single retardation time, or a symmetrical distribution of retardation times, these points appear at the same frequency when plotted against log frequency and would appear at the same temperature when these functions are plotted against temperature, except that there are slight displacements from the variation of Δ*B*, the difference between low and high frequency limiting compliances, with temperature. For an unsymmetrical distribution it can be shown [9] that the following inequalities hold, where *τ*_max_ is the time corresponding to the maximum of bulk retardation spectrum *L_v_*(ln *τ*) defined by the relation,

$$B*=\int_{-\infty }^{\infty }L_{v}(\ln\tau )\frac{1}{1+i\omega \tau }d\ln\tau +B_{\infty },$$

*ω*′ is the angular frequency (*ω*=2*πν*) of the inflection of *B'* versus ln *ω*, and *ω*″ is the frequency of max *B":*

$$\omega^{\prime \prime }>\omega^{\prime }>\frac{1}{\tau_{\max}}$$

if *L_v_*(ln *τ*) falls off more rapidly for *τ*>*τ*_max_ than for *τ<τ*_max_, and

$$\omega^{\prime \prime }<\omega^{\prime }<\frac{1}{\tau_{\max}}$$

if *L_v_*,(ln *τ*) falls off more rapidly for *τ*<*τ*_max_ than for *τ>τ*_max_.

**Figure 4:**
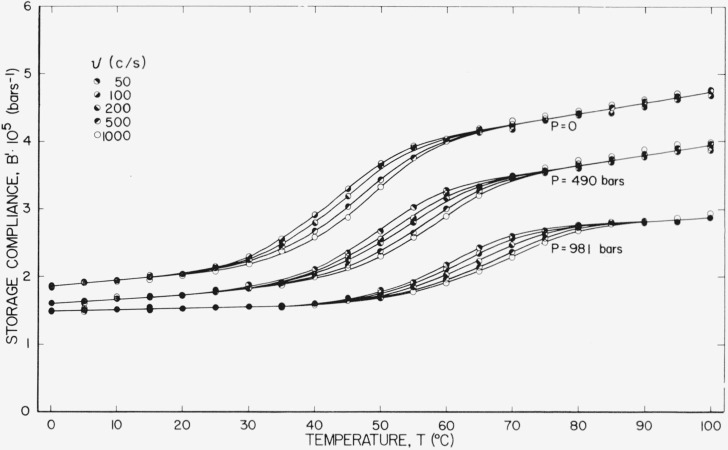
Storage compliance plotted against temperature at several pressures and frequencies.

The storage compliance at different frequencies for three different static pressures is shown in figure 4. The data at 1,000 c/s are identical to that shown in figure 2 under the same conditions. Scatter is higher at the lower frequencies, mostly because of a characteristic 1/*ν* noise [10]. The data at *ν* =50 and *P*=0 were omitted entirely because of excessive scatter.

**Figure 5:**
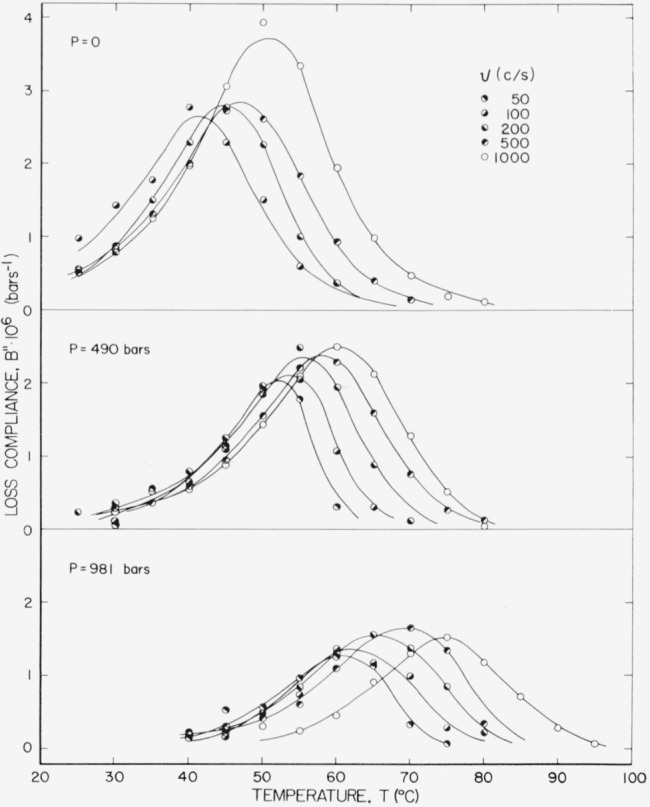
Loss compliance plotted against temperature at several pressures and frequencies.

The corresponding loss compliance is given in figure 5. The maximum values of *B"* at constant pressure generally increase slightly with frequency, which is consistent with the reduced variable formulation discussed in section 5. On the other hand the value of $B_{\max}^{\prime \prime }$ at *v* = 1,000 and *P*=0 is about 25 percent greater than we would expect from extrapolating values at lower frequencies using the reduced variable formulation. A hint of this behavior is seen in figure 3 for *P* = 98 bars also. Whether this behavior is real or an artifact of the experiment cannot be determined from these measurements.

## 5. Reduced Variables

The reduced variable formulation developed for the analysis of our earlier rubber-sulfur data [2] was used. In this treatment the complex bulk compliance,

$$B*(T,P,\omega )=B_{\infty }(T,P)+\Delta B(T,P)ℬ*(T,P,\omega )$$ (1)

is assumed to consist of two parts. The first, *B*_∞_(*T, P*), is the adiabatic compliance,

$$-\frac{1}{V}{\left(\frac{\partial V}{\partial P}\right)}_{s}=-{\left(\frac{\partial \ln V}{\partial P}\right)}_{s},$$

at infinite frequency. The second is the difference between the adiabatic compliances, *B*_0_(*T, P*) at *ω*=0 and *B_∞_*(*T, P*) at *ω*=∞, times a complex normalized dispersion function ℬ*(T,P,ω), which goes to unity at *ω*→0 and zero as *ω*→ ∞.

Assuming that $ℬ*(T^{\prime },P^{\prime },\omega^{\prime })=ℬ*(T,P,\omega )$ (equivalent to an invarient normalized retardation spectrum) when the data are shifted along a temperature, pressure, or log frequency axis, the final equation used to reduce the observed compliances to functions of reduced variables becomes

$$\begin{array}{l} B*(T^{\prime },P^{\prime },\omega^{\prime })=B_{\infty }(T^{\prime },P^{\prime })\\ \;+\frac{\Delta B(T^{\prime },P^{\prime })}{\Delta B(T,P)}[B*(T,P,\omega )-B_{\infty }(T,P)] \end{array}$$ (2)

in which the primes designate either reduced variables or reference conditions. In a reduced variable presentation only one of the independent variables is varied while the others are held constant at the arbitrary reference conditions. *B*_0_ and *B*_∞_ are evaluated empirically from the data in regions where frequency dispersion is insignificant.

The independent variables are reduced following the procedure of Williams, Landel, and Ferry [1] in the development of the WLF Equation with the addition of a linear pressure term in the expression for free volume fraction,

$$f(T,P)=f_{g}+\alpha_{f}(T-T_{g0})-\beta_{f}P(T\geq T_{g}\text{or}P\leq P_{g}),$$ (3)

where *T_g_*_0_ is *T_g_* at *P*=0 and *P_g_* is the glass transition pressure, which may be defined as the break in the volume-pressure curve allowing sufficient time for significant variations to disappear. Frequency and viscosity are related by

$$\omega^{\prime }=\omega \eta (T,P)/\eta (T^{\prime },P^{\prime }),$$ (4)

and viscosity and free volume fraction by

η=Aexp1/f. (5)

The viscosity used here is the steady flow shear viscosity. One can either assume explicitly that the retardation times determining bulk retardations are the same as those involved in shear motions or can look on these relations as defining a separate set of relations for bulk deformations, of the same form as those for shear, evaluating the constants empirically. The limited experimental information available seems to support the hypothesis that the retardation times governing dilatation are the same as those governing shear, except for the complications which may enter in due to the change in volume during the course of a given measurement [11]. In our case the alternating volume changes are too small to introduce any detectable effect of this type.

Equations (3), (4), and (5) are the basic equations necessary to develop the desired relations between the independent variables. Equation (3) written in terms of a viscosity at the arbitrary reference conditions, *T*_0_ and *P*_0_=0 (1 atm), is

$$\eta (T,P)=\eta (T_{0},0)\exp\left[\frac{1}{f}-\frac{1}{f_{0}}\right],$$ (6)

where *f*_0_ is the free volume fraction at the reference conditions. Substituting frequencies for viscosities in eq (6) by utilizing eq (4) with *ω*′ = *ω_r_*, the reduced frequency at the reference conditions, the ratio of corresponding frequencies is

$$\frac{\omega_{r}}{\omega }=\exp\left[\frac{1}{f}-\frac{1}{f_{0}}\right].$$ (7)

Writing eq (7) in logarithmic form and using eq (3) to obtain explicit temperature dependence for *f* and *f*_0_, the final equation for the reduced frequency in terms of the reduced variable parameters, the observed independent variables, *T*, *P*, and *ω*, and the arbitrary reference conditions, *T*_0_ and *P*_0_=0, becomes

$$\ln\omega_{r}=\ln\omega -\frac{\alpha_{f}(T-T_{0})-\beta_{f}P}{\left[f_{g}+\alpha_{f}(T-T_{g0})-\beta_{f}P][f_{g}+\alpha_{f}(T_{0}-T_{g0})\right]}.$$ (8)

This reduces to the WLF Equation, if *T*_0_ is taken as *T_g_* and *P* set equal to zero, for which the “universal” constants correspond to *f_g_*=0.025 and *α_f_*=4.8×10^−4^ °C^−1^.

In a similar fashion the following equivalent expressions are found for a reduced temperature at *P*_0_=0 and *ω*_0_,

$$T_{r}=T-\frac{\beta_{f}}{\alpha_{f}}P+\frac{1}{\alpha_{f}}\frac{{\left[f_{g}+\alpha_{f}(T-T_{g0})-\beta_{f}P\right]}^{2}\ln(\omega_{0}/\omega )}{1-[f_{g}+\alpha_{f}(T-T_{g0})-\beta_{f}P]\ln(\omega_{0}/\omega )},$$ (9)

and a reduced pressure at *T*_0_ and *ω*_0_,

$$P_{r}=P-\frac{\alpha_{f}}{\beta_{f}}(T-T_{0})-\frac{1}{\beta_{f}}\frac{{\left[f_{g}+\alpha_{f}(T-T_{g0})-\beta_{f}P\right]}^{2}\ln(\omega_{0}/\omega )}{1-[f_{g}+\alpha_{f}(T-T_{g0})-\beta_{f}P]\ln(\omega_{0}/\omega )}.$$ (10)

In this formulation the free volume fraction, *f*, is in effect an empirical function of temperature and pressure. It is a free volume fraction related to the difference between *V_l_*, the actual volume in the liquid region, and *V_g_*, the volume obtained by linear extrapolation of the volume-temperature relation established in the glassy region. *α_f_* is generally equal, or very close, to Δ*α*, the difference in isobaric thermal expansivities above and below *T_g_.* If this is assumed,

$$\alpha_{f}={\left(\frac{\partial \ln V_{l}}{\partial T}\right)}_{P}-{\left(\frac{\partial \ln V_{g}}{\partial T}\right)}_{P}={\left(\frac{\partial \ln V_{l}/V_{g}}{\partial T}\right)}_{P}.$$ (11)

An analogous definition of *β_f_*,

$$\beta_{f}=-{\left(\frac{\partial \ln V_{l}/V_{g}}{\partial P}\right)}_{T},$$ (12)

involves the slopes of the isothermal volume-pressure curves below and above the glass transition pressure, *P_g_.* A free volume fraction defined in a manner consistent with these definitions would be

$$f=f_{g}+\ln(V_{l}/V_{g}).$$ (13)

The logarithm of a variable may be expanded in two forms:

$$\ln x=\sum_{n=1}^{\infty }{\left(-1\right)}^{n+1}\frac{{\left(x-1\right)}^{n}}{n}\;0<x\leq 2$$

and

$$\ln x=\sum_{n=1}^{\infty }\frac{{\left(x-1\right)}^{n}}{nx^{n}}.\;x>\frac{1}{2}$$

Representing the logarithm by the first term of the first expansion in eq (13) yields

$$f=f_{g}+\frac{V_{l}-V_{g}}{V_{g}}$$ (14)

and by the first term of the second expansion,

$$f=f_{g}+\frac{V_{l}-V_{g}}{V_{l}}.$$ (15)

The latter is the usual definition of the free volume fraction.

It turns out that (*V_l_–V_g_*)/*V_g_* is a better approximation to ln (*V_l_/V_g_*) for *V_l_*/*V_g_* < 1 and (*V_l_–V_g_*)/*V_l_*, for *V_l_/V_g_* > 1. Since *V_l_/V_g_* is greater than one at temperatures above *T_g_*, (*V_l_–V_g_*)/*V_l_* is the better approximation to ln (*V_l_/V*_g_). This justifies the choice of eq (15) rather than eq (14) as the appropriate approximation to the free volume fraction consistent with the definitions of *α_f_* and *β_f_* given by eqs (11) and (12).

## 6. Evaluation of the Reduced Variable Parameters

### 6.1. Limiting Compliances

The limiting compliances were evaluated as bilinear polynomials in *T* and *P* from a least square fit to the data at temperatures above and below the dispersion region, treating each region separately. Only the 1,000 c/s data were used because they contain the least experimental scatter. Using this procedure the limiting compliances in reciprocal bars are given empirically as:

$$B_{0}(T,P)\times 10^{5}=2.919+1.905\times 10^{-2}T-0.950\times 10^{-3}P-1.011\times 10^{-5}TP,$$ (16)

$$B_{\infty }(T,P)\times 10^{5}=1.865+0.836\times 10^{-2}T-0.433\times 10^{-3}P-0.685\times 10^{-5}TP,$$ (17)

with the difference,

$$\Delta B(T,P)\times 10^{5}=1.054+1.069\times 10^{-2}T-0.517\times 10^{-3}P-0.326\times 10^{-5}TP$$ (18)

in which *T* is in units of °C and *P* in bars.

Δ*B* cannot be measured directly due to the limited frequency range of the apparatus, hence it always involves an extrapolation of either *B*_0_ or *B*_∞_, and sometimes both. The assumption here is that eqs (16) to (18) are valid at all temperatures and pressures within the experimental range, although *B*_0_ was determined from data at temperatures above the dispersion region and *B_∞_*, at temperatures below.

### 6.2. Temperature-Frequency Relations

The range of frequencies covered in these measurements is too small to permit an accurate evaluation of the constants, *f_g_* and *α_f_*, particularly since the calculated shifts are rather insensitive to the exact values of these constants used in the WLF Equation. Our value of Δ*α* at 1 atm is 4.5 × 10^−4^, in good agreement with the value of 4.6×10^−4^ reported by Clash and Rynkiewicz [12]. Ferry [13] finds *α_f_* = 5.9 × 10^−4^ and *f_g_*=0.028 for poly(vinyl acetate), based on the temperature-frequency shifts required to reduce dynamic measurements. The difference between the values of *α_f_* and Δ*α* may be partly due to the fact that both are averages of a sort. *α_f_* is an average value which gives good shifting factors for temperatures above *T_g_*, whereas Δ*α* is an average obtained by approximating two curves, one above and one below *T_g_*, by straight lines.

For convenient reduction of our data we need a single set of constants for use over our whole temperature and pressure ranges. Ferry’s *α_f_*(5.9×10^−4^) times the ratio of our Δ*α* values at 490 bars (our median pressure) and 1 atm (for which pressure Ferry’s *α_f_* was found) gives 4.8×10^−4^ for the value of *α_f_* at 490 bars. Since this corresponds to the “universal” value of *α_f_*, it and the corresponding value for *f_g_*=0.025 were used in reducing our data.

### 6.3. Evaluation of (*∂T/∂P*)*_f_*

(*∂T/∂P*)*_f_* may be determined from the shift of characteristic temperatures (for constant ℬ*) at constant frequency. If the actual frequency range were sufficient, this could be evaluated directly by observing the ratio Δ*T/*Δ*P* necessary to maintain $B_{\max}^{\prime \prime }$ at constant frequency on the loss compliance- frequency plot. The equivalent shift when *B"* is plotted against temperature is the shift of the characteristic temperature, *T*_1_, with pressure at which the following relation (2) is satisfied:

$$\frac{1}{B^{\prime \prime }}\frac{\partial B^{\prime \prime }}{\partial T}=\frac{1}{\Delta B}\frac{\partial \Delta B}{\partial T}.$$ (19)

This is nearly the shift of the temperature of maximum *B"* versus temperature with pressure. The value of (*∂T/∂P*)*_f_* obtained from the shift of the maximum usually agrees within 3 or 4 percent with that from eq (19).

Using the criteria of eq (19) on the curves of figure 3, the characteristic temperature, *T*_1_, is nearly linear with pressure; however, the slope appears to increase slightly with pressure. A least squares fit of *T*_1_ on *P* using all of the curves of figure 3 yields (*∂T/∂P*)*_f_* = 0.024 °C/bar, which is the same as that found for rubber-sulfur using the same criteria. Neglecting the 981 bar curve yields 0.021, which is nearly in agreement with 0.020 which produced the best fit on the reduced variable plots and is consequently used throughout the treatment of these data. It may be that 981 bars is too large for the linear dependence of free volume fraction on pressure assumed in eq (3). In recent work by O’Reilly [14], (*∂T/∂P*)*_f_* was found to be 0.021 from PVT measurements and 0.022 from dielectric measurements (i.e., 0.06 to 10 kc/s).

*α_f_* and *β_f_* are in reasonable agreement with Δ*α* and Δ*β* for many polymers. Differentiation of eq (3) at constant free volume fraction yields

$${\left(\partial T/\partial P\right)}_{f}=\beta_{f}/\alpha_{f},$$ (20)

which should correspond to Δ*β/*Δ*α.* The data of figure 1 are not sufficiently reliable to establish Δ*β* as a function of pressure; however, Δ*B* should be equivalent except that it is assumed to be adiabatic. Table 2 is included to illustrate that although Δ*B* and Δ*α* vary with pressure, the ratio, Δ*B/*Δ*α*, is nearly constant when Δ*B* and Δ*α* are evaluated at temperatures that shift with pressure at a rate of (∂*T*/∂*P*)*_f_.* This ratio does, however, appear to increase slightly with pressure, which is in agreement with the slight increase in ∂*T*_1_/∂*P* with pressure. The obvious distinction between the values of Δ*B/*Δ*α* in table 2 and (∂*T*/∂*P*)*_f_*=0.020 will be discussed in section 8.

## 7. Reduced Variable Presentation

The following presentation includes the reduced data plotted as a function of reduced frequency, reduced temperature, and reduced pressure. The constants used were the WLF Universal Constants given in section 5 and *β_f_/α_f_*=0.020 bars^−1^. All three representations are equivalent because ℬ* is held constant for each shift. They are all included, however, to illustrate the effect of varying each independent variable (*T*, *P*, or *ω*) while the others are held at the reference conditions.

**Figure 6:**
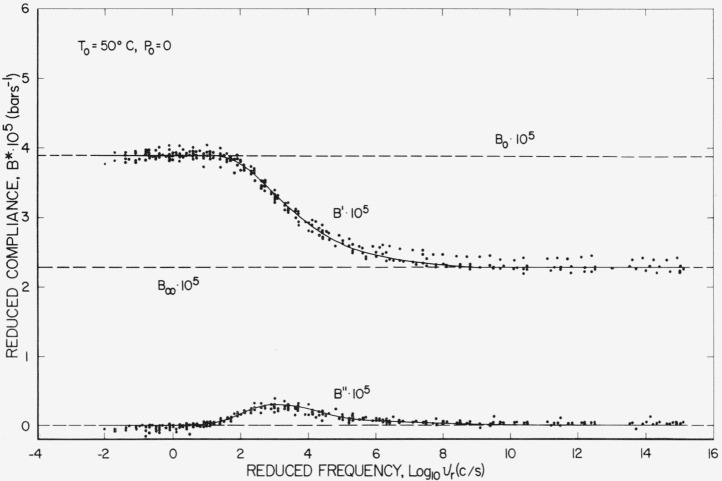
Reduced compliance plotted against reduced frequency referenced to atmospheric pressure and 50 °C.

The simplest reduced variables presentation is a reduced frequency plot referenced to *T*=50 °C and *P*=0 shown on figure 6. This is the only presentation in which the limiting compliances are constants. In figure 6 a distinct set of values of *B'* may be observed on the right which are about 5 percent above *B_∞_.* These values are taken at different frequencies but all at 981 bars, which suggests that at 981 bars the linear dependence of *B*_∞_ on pressure is no longer valid. This distinction may also be observed in the other reduced variable plots presented later. About eight decades are required to cover the dispersion region with a maximum ${ℬ}^{\prime \prime }=0.19$. The corresponding values for a single retardation time would be about 2 decades and ${ℬ}_{\max}^{\prime \prime }$ would be 0.5.

**Figure 7:**
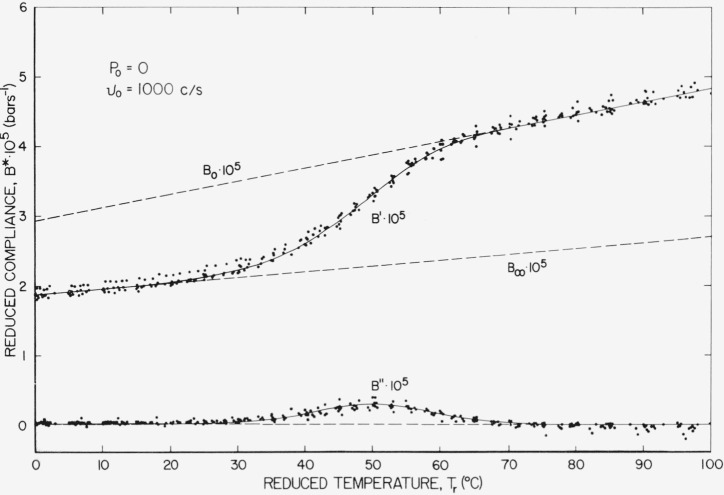
Reduced compliance plotted against reduced temperature referenced to atmospheric pressure and 1,000 c/s.

The reduced temperature plot referenced to *v* =1,000 c/s and *P*=0 is shown in figure 7. This is the most realistic presentation for our data because it involves the least shifting.

**Figure 8:**
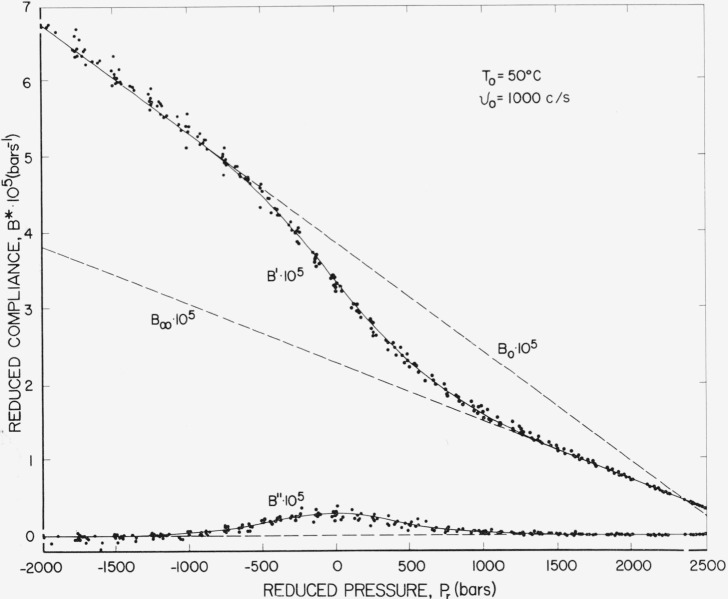
Reduced compliance plotted against reduced pressure referenced to 50 °C and 1,000 c/s.

The reduced pressure plot referenced to *T*=50 °C and *v* = 1,000 c/s is shown on figure 8. This is not a realistic presentation in terms of actual pressure because of the excessive linear extrapolation of the limiting compliances, but does offer interesting suggestions as to the effect of static pressure of the dispersion. Several artifacts appear in this presentation, one being the existence of a negative pressure, which could be avoided by choosing *T*_0_ sufficiently high. Also, the limiting compliances intersect at about 2,300 bars. This again may be due to excessive extrapolation, but does indicate the possible convergence of the limiting compliances at a finite pressure. This would be equivalent to a complete collapse of free volume (at least *f* to *f_g_*) at finite pressure above which frequency dispersion would not be observed.

## 8. Further Discussion

An implication of this free volume concept is that the difference between low and high frequency limiting compliances should be consistent with the horizontal shift of corresponding points, with static pressure, along a temperature plot at constant frequency. That is, the difference between limiting compliances, Δ*B*, should be equal to *β_f_* evaluated from the horizontal shift

$${\left(\partial T/\partial P\right)}_{f}=\beta_{f}/\alpha_{f}$$ (20)

except that Δ*B* is assumed to be adiabatic and *β_f_*, isothermal. Although the observed compliances involve the ratio of alternating volume to alternating pressure, (*∂T/∂P*)*_f_* is a quasi-static phenomenon because it is evaluated from essentially an equilibrium temperature-pressure relationship. Since we have found only an average value for *β_f_*, we should compare this with our Δ*B* evaluated at our median pressure, 490 bars, for which *T*_1_ at 1,000 c/s is 60 °C. Substituting these conditions in eq (18) gives Δ*B*(60,490) = 1.35×10^−5^ bars^−1^. The conversion of this to the corresponding isothermal value may be approximated by the addition of the term *θV_s_α*^2^/*C_p_* [15] where *θ* is the absolute temperature and *C_p_* is the specific heat at constant pressure, to each of *B*_0_ and *B*_∞_ before the difference is taken to obtain Δ*B.* Specific heat data for PVAc apparently do not exist in the literature for pressures other than 1 atm; however, reference 16 gives *C_p_* over a temperature range from 20 to 50 °C at 1 atm for several different rates of heating. Extrapolating limiting heat capacities at slow and fast rates yields 0.43 and 0.32 cal/g °C, respectively. Although these values were not taken at 490 bars, they are considered to be sufficiently reliable to obtain the desired conversion, especially since *α*^2^ is the dominant quantity. Since *α_l_* and *α_g_* change little with temperature, the values *α_l_*=5.65×10^−4^ and *α_g_*=2.03×10^−4^ °C^−1^ taken from table 1 are satisfactory. The observed value of *V*_s_=0.84 cm^3^/g at 60 °C and 490 bars taken from figure 1 was used in both cases because the time average of the specific volume during sinusoidal deformation is appropriate in this connection. Using these values gives for the conversions, *δB*_0_=4.95×10^−6^ and *δB*_∞_=7.6×10^−7^. The difference between these added to Δ*B*=1.35×10^−5^ gives 1.77×10^−5^ for the corresponding isothermal value. The value of *β_f_* calculated from eq (20) using (∂*T*/∂*P*)*_f_*=0.020 °C/bar and *α_f_*=4.8×10^−4^ °C^−1^ (490 bars) is 0.96×10^−5^, which is even smaller than the adiabatic value, Δ*B.*

On the other hand, there was close agreement between *β_f_* and the isothermal value corresponding to Δ*B* for vulcanized natural rubber-12 percent sulfur data [2] using nearly the same criteria for comparison. In this case *β_f_* and the isothermal value corresponding to Δ*B* were 1.15×10^−5^ and 1.21×10^−5^ bars^−1^, respectively.

Another distinction, which is probably related to that above, between PVAc and the rubber-sulfur is the proportion of the compliance of the free volume type to the total given by the ratio Δ*B/B*_0_. For PVAc this was found to be 0.41 compared to 0.25 for the rubber-sulfur, both evaluated at *T_g_*+25 °C and *P*=0.

Although the presence of small amounts of impurities mentioned in section 4.1 influenced the value of the apparent glass transition temperature and possibly the magnitude of the bulk compliances to a slight extent, it is unlikely that the applicability of the free volume concept is strictly limited to very pure polymeric systems. It is conceivable, however, that the excessive value of Δ*B* in PVAc results from additional relaxation processes not present in the rubber-sulfur. For example, with some amorphous polymers, in addition to the alpha process involving changes in configuration of the polymer backbone and therefore associated with the glass transition, contributions to mechanical and dielectric response may result from a beta process involving rotation of side groups along the chain. For polymers that exhibit a beta process the corresponding dispersion region will occur at a lower temperature than that for the alpha process. At low frequencies the two regions of a temperature plot are usually very distinct; however, the different activation energies for the two processes are such that these regions merge at high frequencies and resolution becomes obscured. Since (*∂T/∂P*)*_f_* is a quasi-static phenomenon, one might expect that it would not depend upon the beta process, which would appear unfrozen over our entire experimental range at the long transient times involved, whereas, both dispersion regions might contribute to Δ*B* because of the possibility of these regions merging at the much shorter time scales (corresponding to 1/*ω*) of the alternating pressure.

With PVAc the beta process involves the rotation of the acetate group about the oxygen bond. Dielectric data of Veselovskii and Slusker [17] on PVAc show very distinct loss peaks near 1,000 c/s on a temperature plot for the alpha and beta process at 60 and −80 °C, respectively. These peaks do not appear to merge until well into the megacycle region. These data are fairly consistent with the recent dielectric data of Hikichi and Furichi [18] which give the corresponding temperature for the beta process at −58 °C and a constant apparent activation energy of 10 kcal/mole. Based on this evidence it would be inconsistent to attribute the excessive value of Δ*B* to a contribution resulting from an overlapping beta dispersion region obscured by the alpha process. Aside from the observation that the dispersion regions from the two processes do not merge until within the megacycle region, the beta process is unfrozen over the entire pressure, temperature, and frequency range of our experiment; therefore, its effect should not be observed here except, possibly, in a more complicated fashion than anticipated.

The data of Wada, Hirose, Asano, and Fukutomi [19] suggest a more probable interpretation of this anomaly. Two breaks were observed in their volume-temperature curves for PVAc; one, the alpha or glass transition occurring at 28 °C and a secondary transition at 3 °C. Our volume-temperature data did not reveal the lower break, but it is possible that we did not go to low enough temperatures to include a low temperature change in slope. Wada and coworkers suggest that the low temperature transition is due to a sufficient loosening of free volume to permit rotation of chain ends. Many doubt the validity of such apparent breaks in volume-temperature curves at temperatures below *T_g_* because of uncertainties resulting from the extremely long times required for the physical properties to reach equilibrium; however, even if this break is not real or completely separate from the one at *T_g_*, the interpretation of Wada and coworkers may be justified because of the large temperature range of the glass transition region observed for PVAc.

Wada and coworkers have substantiated the existence of the low temperature transition by observing discontinuities in the complex shear modulus at 33 kc/s for both the 3 and 28 °C transition. Discontinuities in the temperature coefficients of the velocity of sound, dynamic storage moduli, and dynamic storage compliances are often observed with polymers at *T_g_.* These discontinuities, as pointed out by Work [20], are manifestations of the sudden change of thermal expansivity with temperature at *T_g_.* This behavior was, of course, not observed at *T_g_* in our data because such a break would be obscured by the dispersion region. Higher frequencies are necessary to shift the dispersion region on a temperature plot far enough from *T_g_* to observe such discontinuities in the slope of the storage compliance-temperature curve. For a more realistic reduced variable formulation B_∞_ at temperatures above *T_g_* should be evaluated from data at temperatures above *T_g_* and below the dispersion region. Unfortunately this evaluation is not possible with our data for the reason mentioned above. Ultrasonic data on polymers and associated liquids [21] indicate that the slope of the compressibility- temperature curve increases slightly at *T_g_* with increasing temperatures. The corresponding Δ*B* would then be more in agreement with *β_f_* evaluated from (*∂T/∂P*)*_f_*; however, since this correction was not necessary to obtain agreement for rubber-sulfur, it would undoubtedly not be large enough to eliminate the lack of agreement for PVAc.

On the basis of volume-temperature data for PVAc, which show the temperature range of the transition region so large that more than one rate process may be involved, it is conceivable that the dispersion region of the dynamic data is also complicated with additional mechanisms which could result in the large difference between limiting compliances as observed here.

The presence of additional dispersion mechanisms might appear to impose a serious limitation on the reduced variable treatment applied here because of the possibility of different activation energies associated with two or more processes. The assumption in our treatment is that ℬ* is associated with only one type of rate process, namely the alpha process, although many discrete retardation times may be involved. However, since many of the reduced variable parameters are evaluated empirically from the data and contributions from the additional processes are probably small, the treatment applied here is probably legitimate within the experimental error as apparent from a reasonable fit of data in the reduced plots.

After completion of the rubber-sulfur work, it was felt that the free volume concept and corresponding reduced variable treatment applied here was adequate to explain the dynamic compressibility behavior of polymers and possibly some associated liquids. Now that the poly (vinyl acetate) work is complete it is apparent that the reduced variable treatment needs certain refinements to make the parameters appearing in the formulation consistent with corresponding physical constants as postulated by theory. It is planned to continue this work on other systems in order to postulate a better model or make the necessary refinements on our present concepts.

## Figures and Tables

**Table 1:** Thermal expansivities of PVAc for the liquid and glassy states evaluated at T=T*_g_* for each observed pressure

*P*	*T_g_*	*α_l_×*10^4^	*α_g_×*10^4^	Δ*α×*10^4^
				
*Bars*	*°C*	*°C*^−1^	*°C*^−1^	*°C*^−1^
0	17	6.74	2.26	4.48
98	21	6.52	2.21	4.31
245	24	6.19	2.15	4.04
490	26	5.65	2.03	3.62

**Table 2:** *ΔB* at *T*_1_, *Δ*α at *T_g_*, and *ΔB*/*Δ*α at various static pressures

*P*	*T*_1_	*T_g_*	Δ*B*(*T*_1_) ×10^5^	Δ*α*(*T*_g_)× 10^4^	ΔB(*T*_1_)/Δ*α*(*T_g_*)
					
*Bars*	*°C*	*°C*	*Bars*^−1^	°*C*^−1^	*°C/bar*
0	50	17	1.59	4.48	0.035
98	51	21	1.53	4.31	.035
245	56	25	1.48	4.04	.037
490	60	26	1.34	3.62	.037
736	65	…………	1.21	…………	…………
981	74	…………	1.10	…………	…………

## Footnotes

1 Figures in brackets indicate the literature references at the end of this paper.

2 Compliance here is equivalent to compressibility in the usual acoustic usage. This follows the recommendations of the Committee on Nomenclature of the Society of Rheology [8].

3 A solid solution of lead titanate and zirconium titanate supplied by the Clevite Research Laboratory, Cleveland, Ohio.

4 The criterion adopted was that no further change in volume could be observed over a period of 1 hr within the precision of the experiment.
